# Transmission and genomic insights into *Elizabethkingia miricola*: A zoonotic pathogen with intrinsic resistance and nosocomial outbreak potential

**DOI:** 10.1016/j.onehlt.2026.101465

**Published:** 2026-06-04

**Authors:** Shaohua Hu, Xiaohua Meng, Guo Tian, Shujun Ni, Shaojun Hu

**Affiliations:** a State Key Laboratory for Diagnosis and Treatment of Infectious Diseases, National Clinical Research Center for Infectious Diseases, Collaborative Innovation Center for Diagnosis and Treatment of Infectious Diseases, National Medical Center for Infectious Diseases, the First Affiliated Hospital, Zhejiang University School of Medicine, Hangzhou, China; b Yuhang Institute of Medical Science Innovation and Transformation, Hangzhou, China; c Department of Pathology, the First Affiliated Hospital of Zhejiang Chinese Medical University (Zhejiang Provincial Hospital of Chinese Medicine), Hangzhou, China

**Keywords:** *Elizabethkingia miricola*, Genome sequencing, Phylogenetic architecture, Transmission dynamics, Nosocomial outbreak, Resistance mechanisms

## Abstract

*Elizabethkingia miricola* is an emerging opportunistic pathogen with increasing human clinical isolation. Infections pose major therapeutic challenges. This study sequenced the genomes of five clinical *E. miricola* isolates and employed comparative genomics with globally sourced strains to investigate resistance mechanisms, pathogenicity, phylogenetic relationships, and transmission patterns. Phylogenetic analysis revealed that *E. miricola* evolution exhibits no strict correlation with sample source, geographical origin, or region. Isolates from amphibians clustered within clinical lineages, implicating environmental reservoirs—potentially sink water—as infection sources. Geographically, pathogenic *E. miricola* demonstrates worldwide dissemination, with high prevalence in China and Europe. Putative transmission networks indicate intra-hospital spread and transnational circulation, suggesting nosocomial outbreak risks. Shared resistance genes (including *BlaB* and *GOB* β-lactamases) and virulence profiles between human and anuran strains highlight intrinsic resistance and pathogenic potential. Minocycline and doxycycline exhibited potent antimicrobial activity, indicating promising therapeutic alternatives. These findings highlight the zoonotic threat of *E. miricola* via nosocomial and potentially international horizontal transmission. Preventing outbreaks necessitates enhanced genomic surveillance of this pathogen.

## Highlights

• *E. miricola* evolution exhibits no strict correlation with sample source, geographical origin, or region.
• Amphibian-derived isolates clustered within clinical lineages, implicating environmental reservoirs as infection sources.
• Transmission networks indicate intra-hospital spread and transnational circulation, posing nosocomial outbreak risks.
• Shared resistance and virulence profiles underscore *E. miricola's* intrinsic resistance and pathogenic potential.

## 1 Introduction

*Elizabethkingia miricola,* a significant species within the *Elizabethkingia* genus (formerly *Chryseobacterium*), is a non-motile, non-spore-forming, glucose non-fermenting, Gram-negative bacillus (0.5 × 1.0–2.5 μm). It exhibits aerobic metabolism, indole production, and urea hydrolysis, inhabiting diverse natural environments—particularly aquatic and terrestrial ecosystems [1]. First identified in 2003 within condensation water aboard the Russian Mir space station [2]. This bacterium was initially regarded as an uncommon, low-pathogenicity opportunistic pathogen. However, its clinical significance has markedly increased since 2008 [3]. The first documented human infection occurred that year in an immunocompromised patient with mantle cell lymphoma receiving mechanical ventilation in the United States [4]. Subsequent years saw rising reports of invasive nosocomial infections among immunodeficient populations, with manifestations including bacteremia, oral infections, urinary tract infections, pneumonia, sepsis, peritonitis, and meningitis—predominantly affecting immunocompromised adults and neonates in Europe [3], [5], [6], [7], [8], [9], [10], [11]. In 2018, it was first isolated from a cystic fibrosis patient's sputum [12]; in 2020, the initial native joint infection was recorded [13]; and a 2021 case documented fatal intracranial infection, representing the first cerebrospinal fluid isolation of *E. miricola* [14]. Nosocomial outbreaks have escalated recently, with an initial report in 2014 followed by a high-mortality outbreak in Spain that year [15]. Most recently, a 2021 ICU cluster documented 13 infections with a 53.3% case-fatality rate (8 deaths) [16].

Notably, this emerging pathogen demonstrates significant pathogenic potential in amphibian hosts. In 2016, *E. miricola* was first documented as the causative agent of epidemic meningitis-like disease in farmed black-spotted frogs (*Pelophylax nigromaculatus*) in China, with reported mortality exceeding 60% in affected populations [17]. Subsequent phylogenetic analysis revealed close kinship between amphibian-derived isolates and human clinical strains, highlighting potential zoonotic transmission risks [17]. Two years later, Sichuan Province witnessed severe infectious outbreaks in Chinese spiny frogs (*Quasipaa spinosa*), with histopathological examination confirming *E. miricola*-induced multi-systemic lesions [18]. Most recently, Germany reported recurrent outbreaks affecting captive northern leopard frogs (*Lithobates pipiens*) and three additional anuran species [19]. This mounting evidence demonstrates *E. miricola's* capacity for epizootic transmission and underscores its cross-species threat potential to human populations.

Consistently, antimicrobial sensitivity testing reveals a multidrug-resistant (MDR) profile—a ubiquitous phenotype among nearly all previously documented *E. miricola* strains [4], [7], [9], [12], [19], [20], [21]. Treating *E. miricola* infections proves notoriously difficult due to pervasive resistance to numerous antibiotic classes, including extended-spectrum β-lactams and carbapenems [7], [21], [22], [23]. This multidrug resistance, frequent misdiagnosis, and lack of effective therapeutic protocols significantly complicate patient management following infection, often culminating in mortality.

However, research on *E. miricola* remains limited. Literature review indicates nosocomial infections are primarily reported in European countries. Pathogenic *E. miricola* strains are rarely isolated in Asia, particularly China, and their nosocomial transmission and outbreak potential are undetermined. Furthermore, information on this microorganism's evolutionary history, transmission pathways, pathogenesis, and resistance mechanisms is scarce. Genetic diversity among *E. miricola* isolates from various sources and their zoonotic potential are poorly characterized. Understanding its genomic and phylogenetic features would provide crucial insights, yet few *E. miricola* genomes exist in the NCBI database due to historically limited surveillance and clinical whole-genome sequencing. In this study, we isolated five *E. miricola* strains from infected patients. To elucidate the genetic basis of multidrug resistance, pathogenesis, phylogenetic evolution, and transmission, we sequenced these Chinese nosocomial isolates and conducted comprehensive genomic analyses, including comparisons with available global sequences in GenBank. These findings will inform clinical diagnosis, surveillance, prevention, and treatment of *E. miricola*-associated diseases.

## 2 Materials and methods

### 2.1 Study design and bacterial strains

To investigate *E. miricola*, we conducted a systematic screening of clinical *Elizabethkingia* isolates from the Strain Sample Bank at the First Affiliated Hospital of Zhejiang University School of Medicine, yielding five *E. miricola* strains. These isolates, collected between 2013 and 2018 for routine clinical purposes, originated from sputum (*n* = 3), blood (*n* = 1), and urine (n = 1). Initial identification used matrix-assisted laser desorption ionization time-of-flight mass spectrometry (MALDI-TOF MS; Bruker Daltonics), followed by storage as glycerol stocks at −80 °C. Subsequent average nucleotide identity (ANI) analysis confirmed the species designation of our isolates and minimized potential genomic analytical biases. All five strains exhibited ANI values exceeding 95% (Fig. S1), confirming their classification as *E. miricola* per established taxonomic thresholds (> 95% cut-off for ANI) [24]. Additionally, all globally available *E. miricola* genomes within the NCBI database at the time of our study (June 2020) were incorporated (**Table S1**). This study analyzed five newly sequenced strains (2013–2018) alongside 21 public NCBI GenBank genomes, representing diverse clinical, non-clinical, and amphibian-derived sources worldwide (**Table S1**).

### 2.2 Antibiotic susceptibility testing

Antimicrobial susceptibility testing (AST) was conducted using a combined agar dilution and broth dilution approach. Twenty-seven antibiotics were evaluated, with tigecycline, polymyxin, eravacycline, and omadacycline assessed via broth dilution. Susceptibility interpretations (susceptible, intermediate, resistant) followed Clinical and Laboratory Standards Institute (CLSI) guidelines [25], applying the “other non-*Enterobacteriaceae*” criteria except for tigecycline, eravacycline, omadacycline, vancomycin, and rifampin. For tigecycline, eravacycline, and omadacycline—lacking established CLSI or European Committee on Antimicrobial Susceptibility Testing (EUCAST) breakpoints for *E. miricola*—U.S. Food and Drug Administration (FDA) Enterobacteriaceae standards were applied. FDA breakpoints defined as: tigecycline ≤2 μg/mL (susceptible), 4 μg/mL (intermediate), ≥8 μg/mL (resistant); omadacycline ≤4 μg/mL (susceptible), 8 μg/mL (intermediate), ≥16 μg/mL (resistant); eravacycline ≤0.5 μg/mL (susceptible) [26]. Rifampin and vancomycin results were interpreted using CLSI *Enterococcus* species criteria [25]. Quality control utilized *Escherichia coli* ATCC 25922, *Pseudomonas aeruginosa* ATCC 27853, and *Staphylococcus aureus* ATCC 29213 strains.

### 2.3 Genome sequencing assembly and annotation

Overnight cultures of newly isolated strains were incubated in Mueller-Hinton Broth (Oxoid) at 35 °C for 16–18 h. Cells were harvested by centrifugation at 14,000 rpm for 5 min. Genomic DNA was extracted using the Gentra Puregene Yeast/Bact. Kit (Qiagen) according to manufacturer's instructions. DNA integrity was confirmed by agarose gel electrophoresis, with concentration quantified using a Qubit® 2.0 Fluorometer (Thermo Scientific). Sequencing libraries were prepared with the Illumina NEBNext® Ultra™ DNA Library Prep Kit (NEB). Whole-genome sequencing was performed on the Illumina NovaSeq platform (Novogene), producing 150-bp paired-end reads. Raw reads underwent quality filtering to generate clean data, followed by de novo assembly using SOAPdenovo v2.04.

Genome annotation included prediction of coding sequences (Glimmer 3.02 [27], GeneMarkS [28]), transfer RNAs (tRNAscan-SE [29]), ribosomal RNAs (rRNAmmer [30]). Functional annotation of predicted coding sequences was performed using the KEGG database [31].

### 2.4 Comparative genome analysis

Genome similarity among emerging *E. miricola* strains was assessed using FastANI for ANI [32]. Pan-genome and core-genome analyses were performed using CMG-Biotools [33]. Single-nucleotide polymorphism (SNP) phylogenetic tree construction followed established protocols [34]. Briefly, the RedDog pipeline facilitated read mapping, SNP calling, and initial filtering (https://github.com/katholt/RedDog). SNPs common to all *E. miricola* strains were used to generate a maximum-likelihood phylogeny with PhyML [35], rooted via randomized midpoint rooting. Global strain distribution was visualized using the rworldmap package in R (v3.5.3). Minimum spanning trees estimated genetic distances, with strains differing by ≤10 SNPs assigned to identical clusters. Antimicrobial resistance genes were predicted by aligning genomic protein sequences against the Comprehensive Antibiotic Resistance Database (CARD) using RGI [36]. Predictions employed the following thresholds: e-value <1e-30, protein identity >50%, query coverage >50%, subject coverage >50%, match length > 100 amino acids, and identical residues >100 amino acids. Virulence factors were identified using the VFDB Set B database [37] with analogous identity and coverage thresholds. Reproducible workflows for resistance gene analysis (RGI: https://github.com/arpcard/rgi) and virulence factor prediction (VFDB: https://github.com/haruosuz/vfdb) are available.

## 3 Results

### 3.1 Gene repertoire of *E. miricola* species

**Fig. 1:**
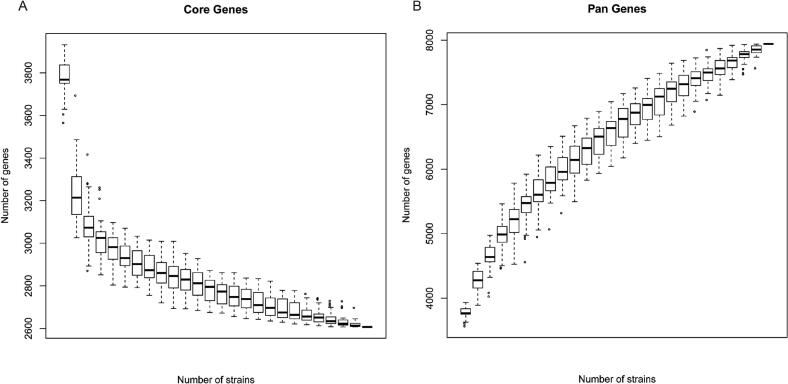
**Core and pan-genome evolution across sampled *E. miricola* genomes.** (A) Core genome size (comprising ubiquitous conserved genes) relative to cumulative genome additions. (B) Pan-genome expansion with incremental genome inclusion.

Core and pan genomes were delineated for selected *E. miricola* strains. Progressive genome analysis revealed a conserved core of 2607 genes alongside 1 to 392 strain-specific genes (Fig. 1). Core genome contraction was observed with incremental genome inclusion (Fig. 1A), while the pan-genome exhibited an open configuration characterized by accessory gene acquisition (Fig. 1B). Notably, strain SKLX011872 harbored 415 unique genes, whereas isolates FL160902, SKLX069005, SKLX070046, and GTC_862 contained merely 1, 2, 5, and 5 strain-specific genes, respectively—indicating substantial genetic homogeneity among these lineages (Fig. 1).

### 3.2 Phylogenetic inference and geographical distribution

**Fig. 2:**
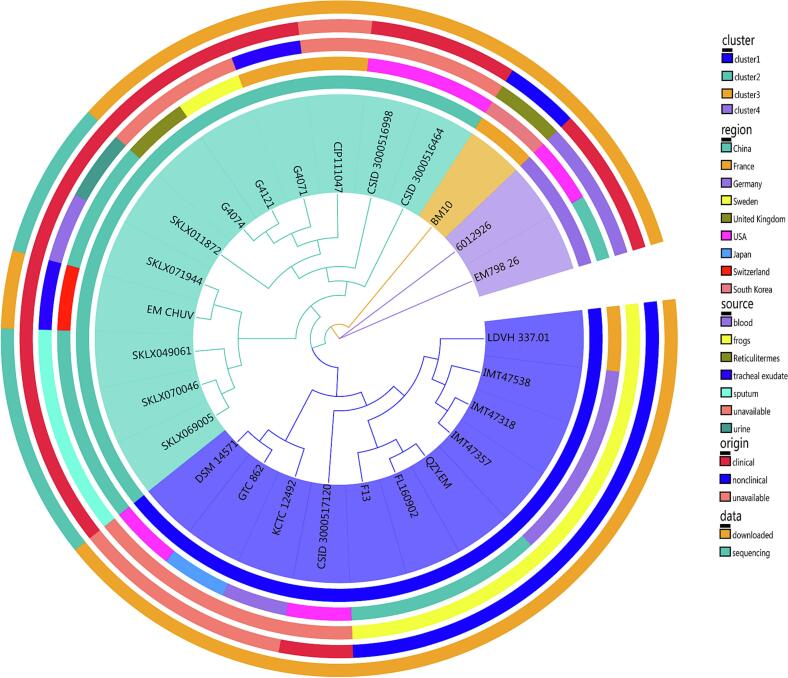
**Phylogenetic architecture of global *E. miricola* genomes based on SNP analysis.** A maximum-likelihood phylogeny shows evolutionary relationships among five novel isolates and twenty-one globally sourced strains. Branches cluster into four color-coded clades (yellow, cyan, blue, purple). Concentric rings depict (inner to outer): phylogenetic cluster, geographic origin, isolation type, clinical status, and source database. Each ring segment corresponds to specific color-coded categories. (For interpretation of the references to color in this figure legend, the reader is referred to the web version of this article.)

To establish a comprehensive genotyping system, we utilized whole-genome sequencing data from 26 global *E. miricola* strains to delineate phylogenetically informative clades and subclades based on SNP architecture. A maximum-likelihood phylogeny constructed from these SNPs revealed distinct branching patterns between most clinical and environmental isolates, with clinical strains exhibiting greater diversity (Fig. 2). Despite substantial genomic variation, the phylogeny segregated predominantly into four major lineages, each containing multiple sub-clusters. Lineage 3 demonstrated the highest heterogeneity, represented exclusively by strain BM10 (Fig. 2). No cohesive phylogenetic groupings with distinct characteristics emerged, indicating evolutionary histories lack significant association with sample source, geographic origin, or region. Notably, clinical isolate CSID 3000517120 showed close evolutionary relationships with environmental strains, including amphibian-derived isolates. Environmental isolates clustering within clinical branches suggest environmental reservoirs—potentially sink water—for *E. miricola* (Fig. 2). These findings suggest possible zoonotic transmission pathways, including amphibian-to-human spread.

**Fig. 3:**
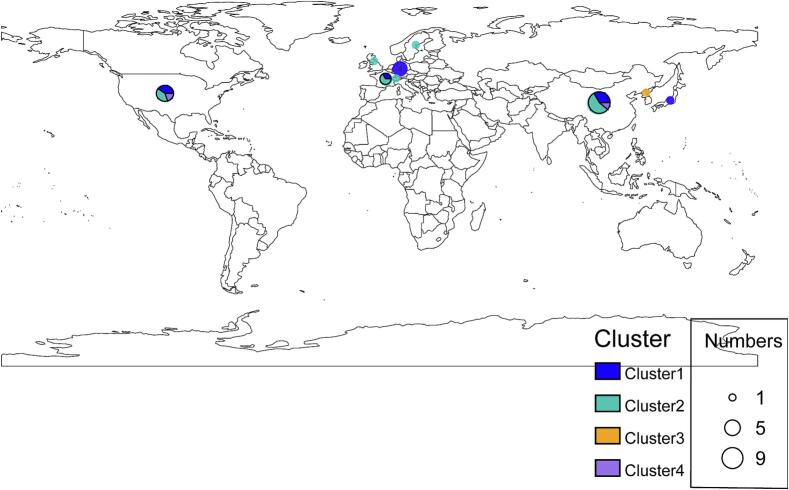
**Geographic distribution of *E. miricola* sampling locations.** The map shows isolate origins. Colored circles represent locations with radii proportional to sample sizes. Pie charts indicate predominant genotype proportions, with colors corresponding to populations defined in Fig. 2.

By integrating genotypic data with geographic origins, we mapped the global distribution of *E. miricola* (Fig. 3). Strains sharing clade affiliation and isolation country were aggregated into representative nodes. This revealed widespread dissemination across multiple nations, with notably high prevalence in China, Germany, and France. Regional economic disparities correlated with genotypic diversity, with Asia exhibiting the greatest heterogeneity. For example, while Asia displayed the most complex evolutionary stratification, Cluster 3 occurred exclusively in South Korea (Fig. 3). *E. miricola* demonstrated a latitudinal distribution pattern, detected across Asia, Europe, and North America but absent from Africa, South America, and Oceania. These findings indicate substantial capacity for cross-border transmission, potentially posing a global epidemiological threat.

### 3.3 Genetic clusters and transmission emergence

**Fig. 4:**
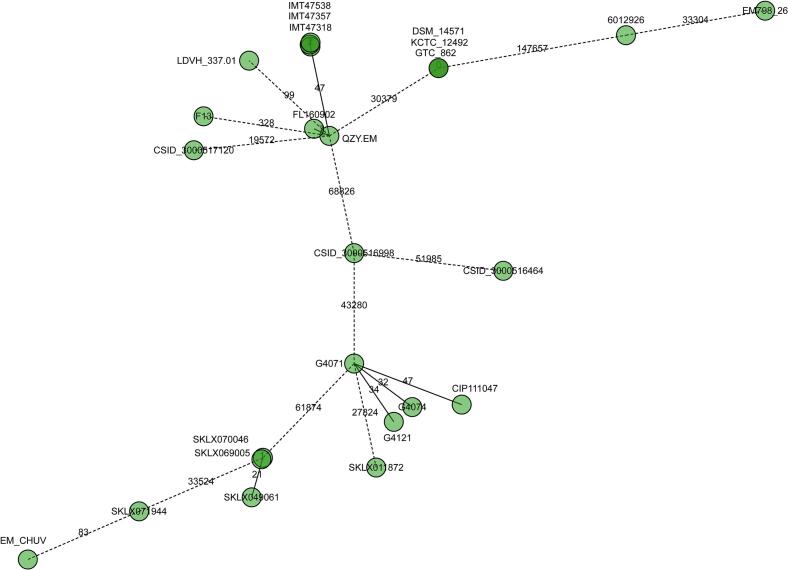
**Transmission network of *E. miricola* isolates inferred from genetic divergence.** Clustering and transmission pathways were reconstructed using minimum spanning trees. Vertices represent isolates and are labeled with sample identifiers. Edges are scaled to indicate SNP-based genetic divergence. Green vertices encompassing multiple strain identifiers denote groups with ≤10 whole-genome SNP differences. Dashed edges, annotated with exact SNP counts, represent greater genetic distances (not to scale). (For interpretation of the references to color in this figure legend, the reader is referred to the web version of this article.)

To investigate *E. miricola* transmission dynamics, we analyzed the proportion of sequenced isolates with genetic linkage (≤10 SNPs). Genotyping 26 isolates identified 20 distinct genotypes (Fig. 4). A star-like topology emerged among pathogens from divergent temporal and geographic origins, suggesting transmission hubs. Within a hypothesized transmission cluster, two strains (G4071 and QZY.EM) showed potential epidemiological links, suggesting a super-spreader role, though requiring validation with expanded clinical data. The network revealed tripartite clustering of isolates IMT47318, IMT47357, and IMT47538—all from German frog specimens despite differing collection sources/dates—implying intra-species transmission. Similarly, clinical isolates SKLX069005 and SKLX070046 formed a distinct cluster; despite different patients, their genomic congruence indicates shared ancestry. Three transnational strains (DSM_14571 [USA], KCTC_12492 [Germany], GTC_862 [Japan]) formed a cluster with near-identical SNP profiles, confirming a common origin (Fig. 4). These networks demonstrate *E. miricola's* capacity for localized hospital transmission and transnational dissemination, underscoring its potential for nosocomial outbreaks.

### 3.4 Antimicrobial susceptibility and resistance determinants

AST results for *E. miricola* isolates are summarized in **Table S2**. These strains exhibited consistent MIC profiles against tested antibiotics, confirming resistance to multiple commonly used agents as previously reported. All isolates demonstrated high-level resistance to at least sixteen antibiotics, establishing their extensively drug-resistant (XDR) status. Resistance encompassed eight antibiotic classes: penicillins, cephalosporins, carbapenems, aminoglycosides, polypeptides, chloramphenicols, tetracyclines, and fosfomycin (**Table S2**). Conversely, levofloxacin, minocycline, doxycycline, and tigecycline showed potent in vitro activity, indicating potential efficacy for clinical *E. miricola* infections.

**Fig. 5:**
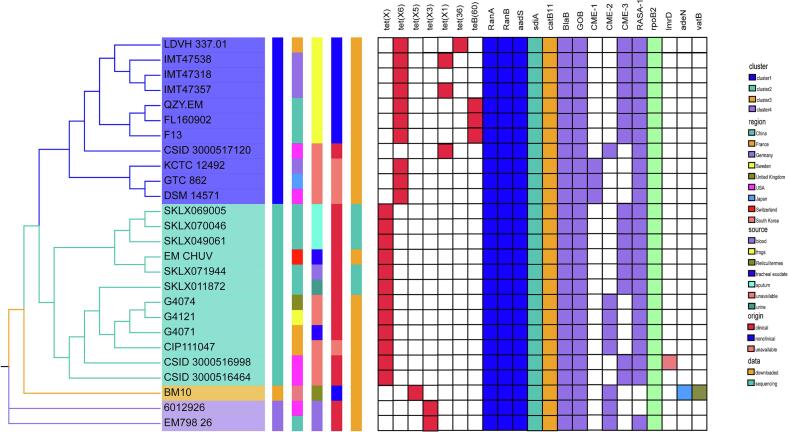
**Distribution of antimicrobial resistance genes in globally sourced *E. miricola*.** The maximum-likelihood phylogenetic tree (left) appears alongside corresponding resistance genotypes (right). Five colored bars adjacent to the phylogeny indicate cluster affiliation, geographic region, sample type, strain origin, and data sources—maintaining chromatic consistency with Fig. 2. Antimicrobial resistance genes clustered by antibiotic class (right panel) are shown as colored boxes denoting their presence in *E. miricola* genomes. Colors correspond to resistance mechanisms: tetracycline, aminoglycoside, fluoroquinolone, chloramphenicol, β-lactam, rifamycin, lincosamide, efflux pump, and streptogramin.

Antibiotic resistance genes were mapped across the *E. miricola* genome collection (Fig. 5). Over twenty distinct acquired antimicrobial resistance (AMR) genes and point mutations were identified. These determinants primarily conferred resistance to tetracyclines, aminoglycosides, fluoroquinolones, beta-lactams, chloramphenicol, and efflux mechanisms. AMR profiles showed minimal lineage-specific variation. Only lincosamide resistance (*lmrD*), streptogramin resistance (*vatB*), and an efflux pump gene (*adeN*) exhibited variable presence among related reticulitermes and environmental isolates (Fig. 5). Resistance gene distribution lacked correlation with geographical origin or isolation source, indicating substantial homogeneity among sepsis-derived isolates. All genomes harbored intrinsic carbapenemase genes *BlaB* and *GOB*, cephalosporin resistance gene *CME*, three aminoglycoside resistance genes (*RanA, RanB, aadS*), and fluoroquinolone resistance gene *sdiA*, confirming species-wide intrinsic resistance to these classes. Strong genotype-phenotype concordance elucidated resistance mechanisms, particularly to carbapenems and cephalosporins.

### 3.5 Virulence genes and potential pathogenicity

**Fig. 6:**
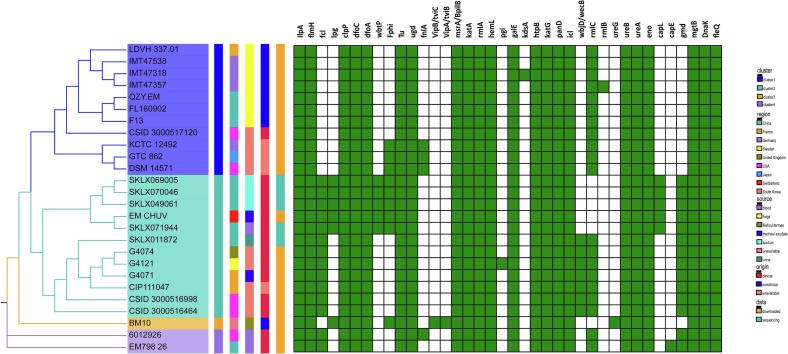
**Phylogenetic distribution of virulence-associated genes.** A maximum-likelihood phylogeny of 26 *E. miricola* isolates with metadata (genotype, geographical origin) appears alongside virulence gene profiles. Phylogenetic reconstruction and color annotations (left) correspond to Fig. 5. Virulence gene distribution (right) shows known genes as green (present) or white (absent) rectangles. (For interpretation of the references to color in this figure legend, the reader is referred to the web version of this article.)

The pathogenic mechanisms of *Elizabethkingia* species remain largely unknown. Bacterial pathogenicity depends on the presence and expression of virulence-associated genes. Genomes of collected *E. miricola* isolates were analyzed for known virulence factor (VF) determinants. Homologs of established virulence factors were identified in these isolates. This study detected up to 38 distinct VF categories in selected *E. miricola* strains (Fig. 6). These VFs contribute to essential biological processes including capsule polysaccharide synthesis, flagellar and fimbrial motility, lipopolysaccharide/lipid A biosynthesis and metabolism, desferrioxamine transport and metabolism, heme biosynthesis, stress adaptation (catalases, peroxidase, heat shock proteins), and two-component regulatory systems (Fig. 6). They also encode toxins, invasion factors, antiphagocytic elements, secretion systems, and hemolysins. Results reveal a predominance of genes encoding adherence factors, secretion systems, and iron acquisition mechanisms.

The conserved VFs universally present across genomes strongly suggest *E. miricola* may be an emerging inherent pathogen. For instance, the heme biosynthesis and utilization gene *hemL* was detected in all isolates, partially explaining the bacterium's hematogenous pathogenicity. Similarly, genes associated with flagellar and fimbrial motility, and capsule polysaccharide synthesis were universally identified, indicating adhesive and invasive capabilities. Although 22 virulence-associated genes were conserved among all studied strains (Fig. S2), lineage-specific patterns with minimal intra-lineage variation were observed. Notably, the five characterized isolates possessed a greater virulence gene repertoire than other strains (Fig. 6), suggesting enhanced pathogenicity and significant research value. While minor variations exist between clinical and environmental isolates, virulence gene profiles show minimal correlation with strain source or geographic origin, supporting *E. miricola's* intrinsic pathogenicity.

## 4 Discussion

*E. miricola* is ubiquitous in natural and clinical environments, including soil, wastewater, and healthcare facilities. Historically considered an uncommon opportunistic pathogen with low virulence, it has recently emerged as a significant life-threatening human pathogen. A 2021 report documented 13 cases in a single Spanish ICU, confirming its outbreak-associated opportunistic nature with a 53.3% mortality rate [16]. Previous research has focused primarily on case reports and antimicrobial susceptibility profiles, while comprehensive genomic data remain limited. Existing genomic analyses predominantly involve environmental or amphibian-derived strains. This study addresses this gap through complete genome sequencing and comparative genomic analysis of five clinical *E. miricola* isolates from China. To our knowledge, this constitutes the first extensive analysis of evolutionary relationships and genomic comparisons among *E. miricola* strains using a comprehensive set of clinical, environmental, and amphibian-derived genomes.

The true incidence of *E. miricola* infection is likely underestimated. Before 2024, only six cases had been documented [11]. This emerging pathogen is frequently misidentified as other *Elizabethkingia* species or dismissed as contamination. Phenotypic methods cannot differentiate *E. meningoseptica*, *E. miricola*, and *E. anophelis*, leading to misdiagnosis or dismissal as contaminants due to insensitive clinical detection systems [38], [39], [40]. For example, a postoperative pulmonary infection case experienced delayed appropriate antimicrobial therapy following initial *E. miricola* misidentification [11]. Early diagnostic efforts for this patient proved inadequate: Computed tomography (CT) findings were inconclusive, and sputum smear/culture failed to detect the pathogen, resulting in inappropriate empirical ceftizoxime treatment [11]. Historically, 16S rRNA gene sequencing—considered the gold standard—has identified most clinical isolates [41]. However, distinguishing *Elizabethkingia* species by 16S rRNA alone is challenging due to five nearly identical gene copies within the genus [42], necessitating alternative identification methods. The highly specific *rpoB* and *mutT* genes serve as effective biomarkers for definitive *E. miricola* identification via qPCR, showing no cross-reactivity with other bacteria [23], [43]. However, this PCR method is laborious and costly. Consequently, several rapid, sensitive detection methods have recently emerged, including recombinase polymerase amplification combined with lateral flow dipstick (RPA-LFD) and fluorescent probe-based recombinase polymerase amplification (exo RPA), both enabling detection within 30 min [44]. Fourier-transform infrared (FTIR) spectroscopy, a real-time bacterial typing technology, has proven effective for rapid outbreak identification [15]. Additionally, culture-independent meta-genomic next-generation sequencing (mNGS) represents a robust method for identifying this infrequently isolated pathogen [38]. Notably, our investigations revealed initial MALDI-TOF MS misidentification of two *E. meningoseptica* and three *E. miricola* isolates, all ultimately reclassified as *E. miricola* through genomic sequencing and alignment.

*E. miricola* is an emerging pathogen presenting significant therapeutic challenges due to intrinsic extensive antibiotic resistance [40]. As established, *Elizabethkingia* species typically resist carbapenems, cephalosporins, aminoglycosides, tetracyclines, and colistin, while showing variable susceptibility to piperacillin-tazobactam, fluoroquinolones, and trimethoprim-sulfamethoxazole [45]. Our findings confirm levofloxacin and ciprofloxacin efficacy, though trimethoprim-sulfamethoxazole showed limited activity. However, empirical fluoroquinolone use remains controversial, given documented resistance in *E. miricola* urinary tract infections [11], [46]. Minocycline consistently demonstrates efficacy against *Elizabethkingia* species [45], [47], with all isolates in this study exhibiting high susceptibility. Doxycycline also proved highly effective, establishing its utility for clinical empirical therapy. Tigecycline activity warrants further investigation: while our isolates were susceptible, prior studies reported only 88.46% susceptibility among isolates [15]. This extensive resistance, combined with limited empirical options, complicates adequate antibiotic coverage, highlighting the need for alternative regimens. Notably, Chinese herbal medicines represent a promising strategy, with *Caesalpinia sappan* and *Rhus chinensis* extracts showing potent activity against *E. miricola* (MIC <0.2 mg/mL) [48].

Several limitations warrant acknowledgment in this study. Although comprehensive clinical, environmental, and amphibian-derived *E. miricola* isolates were analyzed, the collection may lack adequate global representativeness and standardized characterization. Enhancing sample diversity and regional coverage should be expanded in subsequent research. While concordant genotypic and phenotypic results reveal potential antibiotic resistance mechanisms, these cannot be fully determined solely through phenotypic manifestations. Definitive characterization requires targeted mechanistic validation studies, such as gene editing to confirm expression effects.

## 5 Conclusions

In summary, *E. miricola* is confirmed to be a significant pathogenic bacterium capable of causing life-threatening human infections. The conserved distribution of AMR genes and VFs across isolates from diverse sources demonstrates this pathogen's inherent multidrug resistance and intrinsic pathogenicity. Our findings preliminary demonstrate *E. miricola's* potential as a zoonotic pathogen with capacity for nosocomial and international dissemination, even presenting a global transmission threat. These results provide foundational insights for formulating targeted disease control and prevention strategies against this emerging pathogen.

The following are the supplementary data related to this article.

Figure S1 **Heatmap showing average nucleotide identity (ANI) values among representative *E. miricola* isolates.**
Figure S2. **Prevalence distribution of virulence-associated genes.**

Supplementary Table S1 *E. miricola* isolates and comparative strains included in the study.

Supplementary Table S2 The antimicrobial MICs (mg/L) of 5 *E. miricola* isolates determined using the agar/broth dilution.

## CRediT authorship contribution statement

**Shaohua Hu:** Writing – original draft, Methodology, Data curation, Conceptualization. **Xiaohua Meng:** Software, Formal analysis, Data curation. **Guo Tian:** Visualization, Project administration, Formal analysis. **Shujun Ni:** Visualization, Resources, Investigation. **Shaojun Hu:** Writing – review & editing, Supervision, Funding acquisition, Conceptualization.

## Ethics approval and consent to participate

This study was conducted in accordance with the Declaration of Helsinki and approved by the Clinical Research Ethics Committee of the First Affiliated Hospital, Zhejiang University School of Medicine. As this surveillance constituted routine infection control without presentation of identifiable human data, the requirement for participant consent was waived.

## Funding

This research was supported by the 10.13039/501100001809National Natural Science Foundation of China (No.82302589), 10.13039/100005622the Medical and Health Research Project of Zhejiang Province (No.2024KY948).

## Declaration of competing interest

The authors declare that they have no conflict of interest.

## Data Availability

The genome sequences of the five newly assembled *E. miricola* strains are deposited in the NCBI GenBank database under BioProject accession number PRJNA739046. Individual genomes are accessible under their respective accession numbers.
