# Supplementary material for: Transmission and genomic insights into Elizabethkingia miricola: A zoonotic pathogen with intrinsic resistance and nosocomial outbreak potential
Source: One Health. 2026 Jun 4;22:101465. doi: 10.1016/j.onehlt.2026.101465 (PMC13264240; doi:10.1016/j.onehlt.2026.101465)
Supplement: Figure S1 — Heatmap showing average nucleotide identity (ANI) values among representative E. miricola isolates. Figure S2. Prevalence distribution of virulence-associated genes. [file mmc1.pdf]

Supplemental Figures

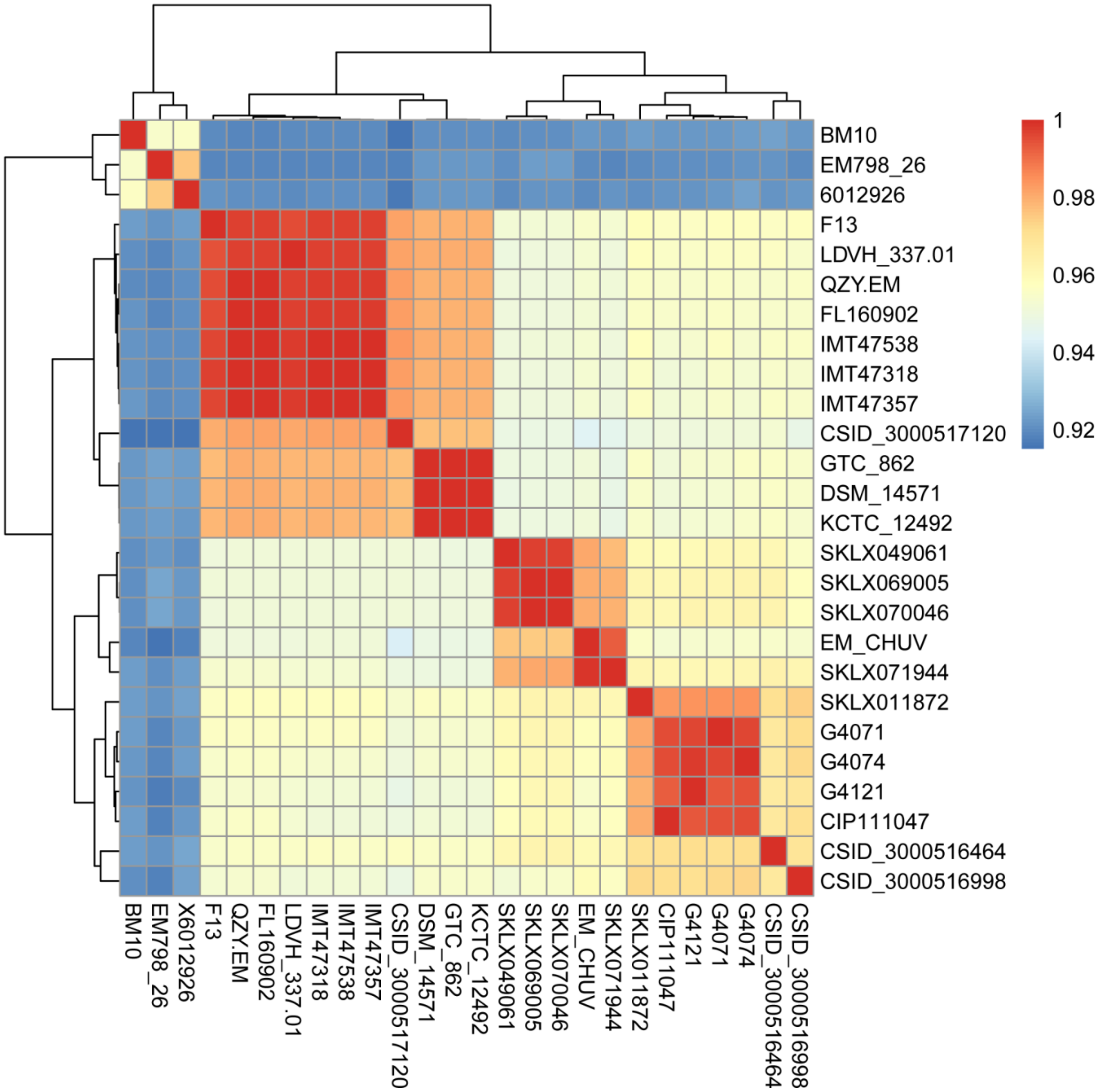

Figure S1. Heatmap showing average nucleotide identity (ANI) values among representative *E. miricola* isolates. The analysis includes 26 strains comprising five newly sequenced isolates from this study and 21 public genomes.

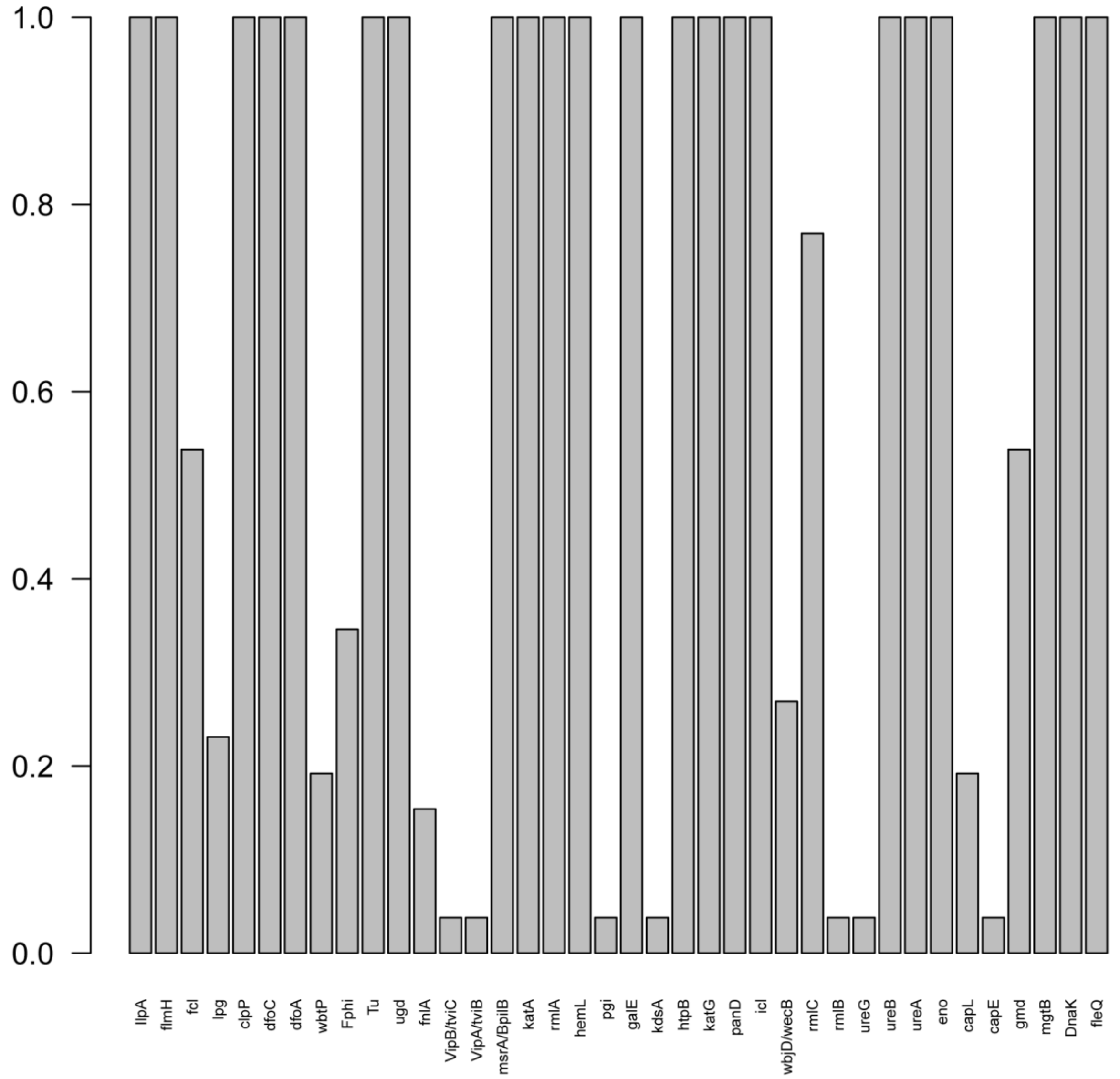

Figure S2. Prevalence distribution of virulence-associated genes. The x-axis shows virulence gene identity, and the y-axis indicates the proportion of isolates carrying each gene. This profile corresponds to Figure 6.
